# Antioxidants protect against diabetes by improving glucose homeostasis in mouse models of inducible insulin resistance and obesity

**DOI:** 10.1007/s00125-019-4937-7

**Published:** 2019-07-15

**Authors:** Leon G. Straub, Vissarion Efthymiou, Gerald Grandl, Miroslav Balaz, Tenagne Delessa Challa, Luca Truscello, Carla Horvath, Caroline Moser, Yael Rachamin, Myrtha Arnold, Wenfei Sun, Salvatore Modica, Christian Wolfrum

**Affiliations:** 1 Laboratory of Translational Nutrition Biology, Institute of Food, Nutrition and Health, Swiss Federal Institute of Technology, ETH Zürich, CH-8603 Schwerzenbach, Switzerland; 2 Touchstone Diabetes Center, UT Southwestern Medical Center, Dallas, TX USA; 3 Institute for Diabetes and Obesity, Helmholtz Diabetes Center, Helmholtz Zentrum München, Neuherberg, Germany

**Keywords:** Acetovanillone, Adipocyte, Adipocyte quantification, Adipocyte-specific, Adipose tissue, Antioxidants, Apocynin, CreERT2, Diet-induced obesity, Fat, Hyperglycaemia, Hyperinsulinaemic–euglycaemic clamp, Hyperphagia, iFIRKO, Insulin receptor, Insulin resistance, Leptin deficiency, Lipolysis, *N*-acetylcysteine, *ob/ob*, Obesity resistance, Polydipsia obesity, Tamoxifen, Type 2 diabetes

## Abstract

**Aims/hypothesis:**

In the context of diabetes, the health benefit of antioxidant treatment has been widely debated. In this study, we investigated the effect of antioxidant treatment during the development of insulin resistance and hyperphagia in obesity and partial lipodystrophy.

**Methods:**

We studied the role of antioxidants in the regulation of insulin resistance using the tamoxifen-inducible fat-specific insulin receptor knockout (iFIRKO) mouse model, which allowed us to analyse the antioxidant’s effect in a time-resolved manner. In addition, leptin-deficient *ob/ob* mice were used as a hyperphagic, chronically obese and diabetic mouse model to validate the beneficial effect of antioxidants on metabolism.

**Results:**

Acute induction of insulin receptor knockout in adipocytes changed the substrate preference to fat before induction of a diabetic phenotype including hyperinsulinaemia and hyperglycaemia. In healthy chow-fed animals as well as in morbidly obese mice, this diabetic phase could be reversed within a few weeks. Furthermore, after the induction of insulin receptor knockout in mature adipocytes, iFIRKO mice were protected from subsequent obesity development through high-fat diet feeding. By genetic tracing we show that the persistent fat mass loss in mice after insulin receptor knockout in adipocytes is not caused by the depletion of adipocytes. Treatment of iFIRKO mice with antioxidants postponed and reduced hyperglycaemia by increasing insulin sensitivity. In *ob/ob* mice, antioxidants rescued both hyperglycaemia and hyperphagia.

**Conclusions/interpretation:**

We conclude that fat mass reduction through insulin resistance in adipocytes is not reversible. Furthermore, it seems unlikely that adipocytes undergo apoptosis during the process of extreme lipolysis, as a consequence of insulin resistance. Antioxidants have a beneficial health effect not only during the acute phase of diabetes development, but also in a temporary fashion once chronic obesity and diabetes have been established.

**Electronic supplementary material:**

The online version of this article (10.1007/s00125-019-4937-7) contains peer-reviewed but unedited supplementary material, which is available to authorised users.

## Introduction

Type 2 diabetes is defined as a metabolic disorder characterised by systemic insulin resistance which results in hyperglycaemia. Epidemiological studies identified high BMI as a risk factor for developing diabetes [1]. Insulin resistance of adipose tissue leads to increased plasma NEFA levels through aberrant regulation of lipolysis. In humans, lipoatrophy was shown to drive systemic insulin resistance [2], while disruption of the insulin signalling cascade in adipocytes in mice causes lipoatrophy and promotes the development of type 2 diabetes [3, 4]. Thus, it has been proposed that elevated NEFA concentrations in blood are the primary cause of insulin resistance through lipid accumulation in non-adipose tissues [5]. In recent years, a new generation of genetic insulin receptor (IR) knockout mouse models enabled researchers to establish the causal link between adipose tissue insulin resistance and the development of type 2 diabetes [3, 6]. The tamoxifen-inducible fat-specific IR knockout (iFIRKO) mouse model established for the first time a causal relationship between insulin resistance in adipocytes and early stages of type 2 diabetes development [6]. Interestingly, IR ablation in adipocytes not only promotes lipolysis but also reduces blood leptin concentrations, which has been suggested to cause the hyperglycaemic phenotype of iFIRKO mice. Leptin informs the brain on the body’s energy reserves [7]. Low blood leptin concentrations indicate imminent depletion of fat stores and trigger responses that aim at acquiring and preserving energy [8]. In consequence, congenital leptin deficiency causes overeating which leads to obesity in early life in humans [9, 10], as well as in mice [11]. Another factor reported to contribute to insulin resistance in the adipose tissue is oxidative stress [12–14]; however, this concept has been widely criticised for the lack of evidence and negative outcomes in both human correlative food supplementation studies and mouse experiments [15].
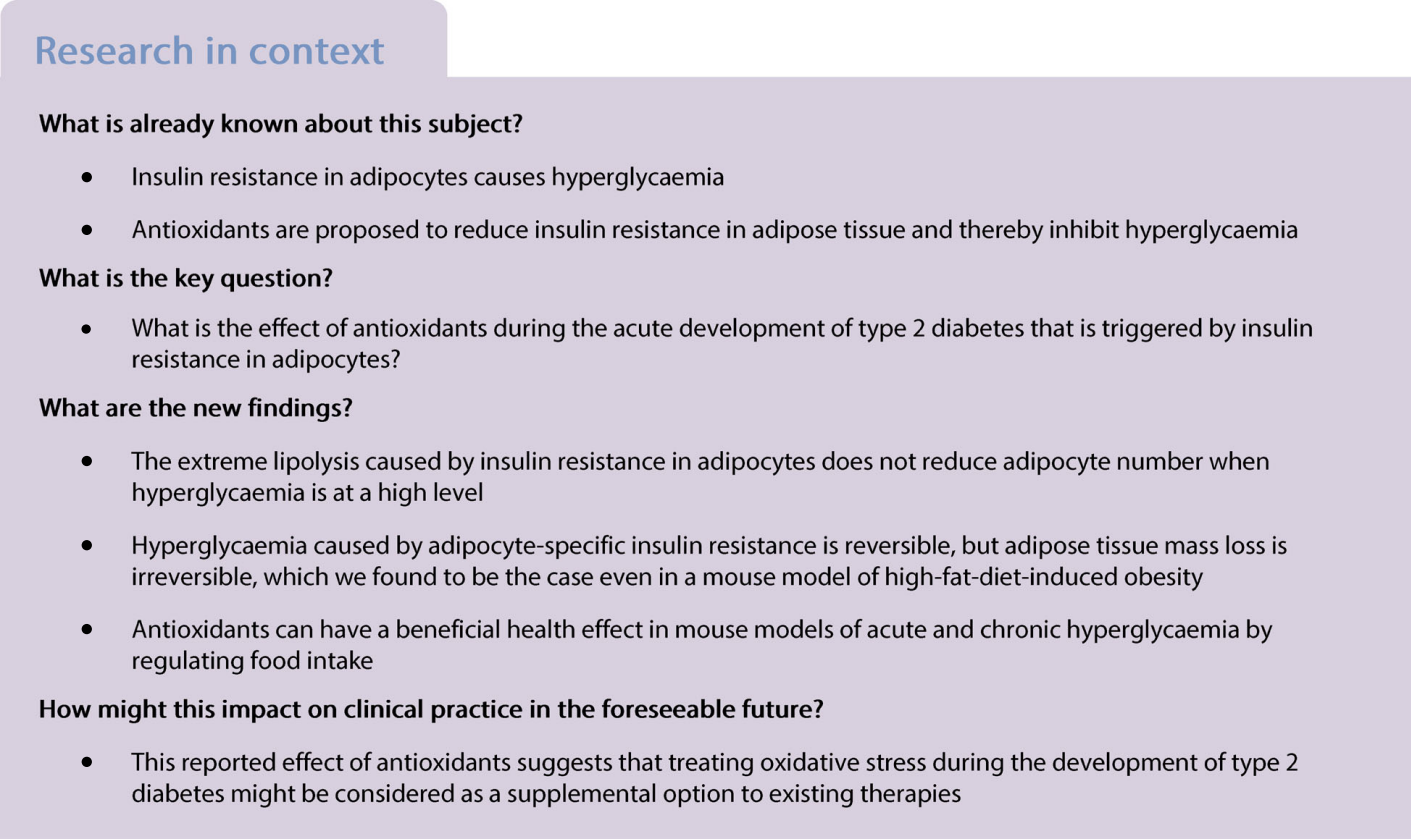


## Methods

### Animal work

*Adipoq*-CreERT2 animals were created by bacterial artificial chromosome (BAC) cloning of CreERT2 into the RPCI-24-69M4 BAC vector (BACPAC, Oakland, CA, USA) and pronuclear injection. iFIRKO mice were created by breeding *Adipoq*-CreERT2 with *IR*^fl/fl^ mice (B6.129S4(FVB)-*Insr*^tm1Khn^/J) [16], which had been back-crossed to C57BL6 mice for 10 generations. Breeding with *Rosa26*-tdRFP mice (*Gt(ROSA)26Sor*^tm1Hjf^) [17] resulted in the iFIRKO-chaser mouse (loxPStoploxP-tdRFP transgene). Male mice of 10–14 weeks of age were used for all experiments and unless stated otherwise were housed at 23°C on an inverted light cycle. *ob/ob* (B6.Cg-*Lep*^*ob*^/J) mice were purchased from Jackson Laboratory (Bar Harbor, ME, USA). Breeding and experiments were performed in the SLA-Schwerzenbach animal facility of ETH Zurich. Feeding a high-fat diet (HFD) with 60% of energy derived from fat (purified diet #2127; Kliba-Nafag, Kaiseraugst, Switzerland) induced obesity. CreERT2 activity was induced by gavage of 2 mg tamoxifen (Sigma-Aldrich, St. Louis, MO, USA) per mouse in 100 μl sunflower oil for 3 consecutive days. The antioxidants *N*-acetylcysteine (NAc) and apocynin (also known as acetovanillone) were dissolved in drinking water at concentrations of 15 mmol/l and 40 mmol/l, respectively. Body composition was measured with NMR scanning (EchoMRI, Houston, TX, USA). GTT and ITT were performed after a 4–5 h fast. Fasting blood glucose was measured from the tail vein with ACCU-Check Aviva Blood Glucose Meter System (Roche, Basel, Switzerland). Glucose solution (1 g glucose per kg body weight, in 0.9% NaCl, Braun, Kronberg, Germany) or insulin solution (0.6, 1, 1.5 or 2 U insulin Actrapid HM per kg body weight, Novo Nordisk [Bagsværd, Denmark], in 0.9% NaCl) was injected intraperitoneally. All experiments were performed according to national and institutional guidelines, which are in line with Animal Research: Reporting of In Vivo Experiments (ARRIVE) guidelines and the EU directive 2010/63/EU.

### Indirect calorimetry

Indirect calorimetry was performed with a metabolic cage system (PhenoMaster, TSE Systems, Bad Homburg, Germany). O_2_ consumption ($\dot{V}{\mathrm{O}}_2$) and CO_2_ production ($\dot{V}{\mathrm{CO}}_2$) were calculated using TSE PhenoMaster V5.6.5 with corresponding coefficients of 3.941 (C$\dot{V}{\mathrm{O}}_2$) and 1.106 (C$\dot{V}{\mathrm{CO}}_2$). Respiratory exchange ratio (RER) was calculated as the ratio of $\dot{V}{\mathrm{CO}}_2$ to $\dot{V}{\mathrm{O}}_2$. Mice were acclimated to the system for 24 h before measurements.

### Blood plasma content measurement

Insulin concentration was measured using the Mouse/Rat Insulin kit (Meso Scale Discovery, Rockville, MD, USA). NEFA levels were measured using the NEFA-C kit (Wako Chemicals, Neuss, Germany). Leptin concentrations were measured with the leptin Mouse/Rat ELISA (BioVendor, RD291001200R, Heidelberg, Germany) and T_4_ concentrations with the T_4_ ELISA kit (Invitrogen, EIAT4C, Carlsbad, CA, USA).

### Hyperinsulinaemic–euglycaemic clamp

Before the hyperinsulinaemic–euglycaemic clamp, mice were fasted for 5 h. The surgery as well as overall clamp procedure were performed as previously published [18].

### Western blots

IR-β subunit (Santa Cruz Biotechnology, #SC-711, Dallas, TX, USA) and Pan-Actin (Cell Signaling Technology, #8456, Danvers, MA, USA), were used in western blots. Band intensity was quantified using the ImageJ 1.52a function ‘Analyse→Measure’. The western blot protein band signal was calculated as ([integrated density of IR-β / integrated density of Pan-Actin] − background signal) normalised to the *IR*^fl/fl^ sample.

### mRNA analysis

Total RNA was isolated with TRIzol (Invitrogen, Carlsbad, CA, USA), then reverse-transcribed with the High Capacity cDNA Reverse Transcription kit (Applied Biosystems, Foster City, CA, USA). Sybr Green quantitative PCR (qPCR) was used (Thermo Fisher Scientific, Waltham, MA, USA). Primer sequences are listed in electronic supplementary material (ESM) Tables 1, 2. Gene expression was referenced to that of TATA-box binding protein (encoded by *Tbp*).

### Quantification of Cre recombination in mouse tissues

Tissue was homogenised in 1 ml of 50 mmol/l NaOH (Sigma-Aldrich) with Thermolyser LT (Qiagen, Hilden, Germany) and 250 μl of 1 mmol/l Tris/HCl (Sigma-Aldrich) was added to neutralise the pH. Samples were centrifuged twice at 12,000 *g* for 5 min and the aqueous phase was transferred to a fresh tube. To quantify labelled adipocytes, primers (ESM Table 2) were designed to identify recombined loxP sites in tandem red fluorescent protein (tdRFP) transgene (loxPStoploxP-tdRFP) [17], which reflects the amount of adiponectin-positive cells. Apolipoprotein B (ApoB) was used for total cells. Absolute recombined loci were quantified by quantitative PCR of genomic DNA using a standard curve generated from synthesised plasmid pUC57recloxPRFP-ApoB.

### Statistical analysis

All results are expressed as mean ± SEM; all graphics and statistical analyses were performed using GraphPad Prism 7. Statistical significance was calculated using multiple two-tailed unpaired Student’s *t* tests, or two-way ANOVA with Sidak’s multiple comparisons test. Statistical significance is indicated as: *p < 0.05, **p < 0.01, ***p < 0.001.

## Results

### iFIRKO mice show impaired glucose homeostasis

**Fig. 1:**
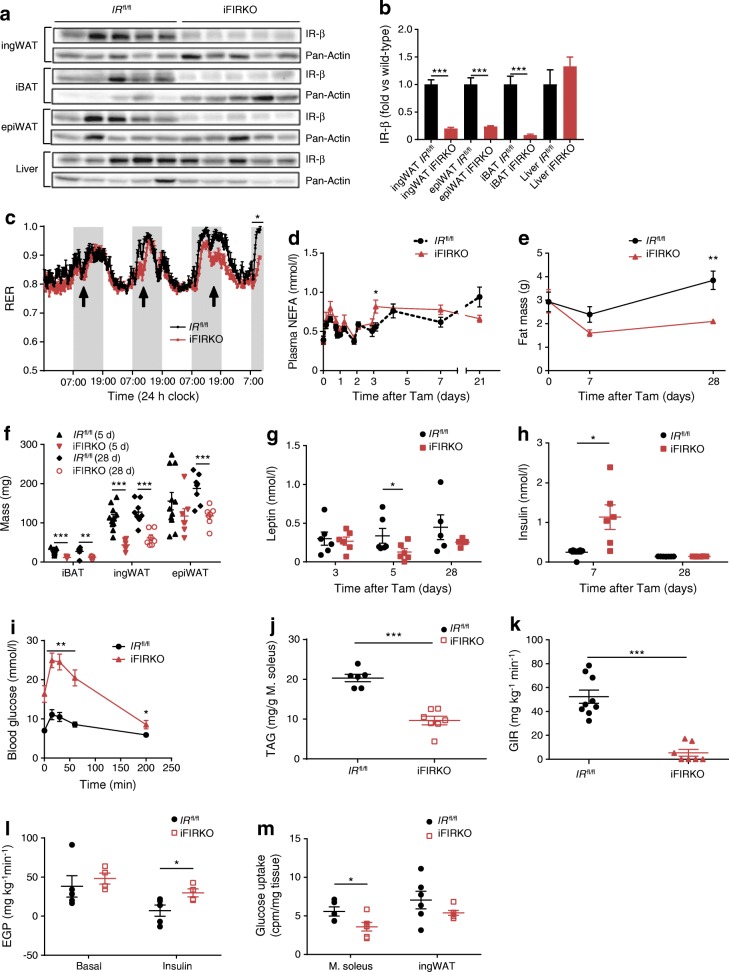
Inducible fat-specific IR knockout first reduces RER then leads to hyperinsulinaemia that correlates with hypoleptinaemia and insulin resistance, before it reduces the mass of all adipose tissue depots. (**a**) Western blot of IR-β and Pan-Actin in adipose tissue and liver lysates (*n* = 5 for *IR*^fl/fl^ and iFIRKO). (**b**) Quantification of western blot band intensity of IR-β normalised to Pan-Actin and expressed as fold vs wild-type (*IR*^fl/fl^) in adipose tissue and liver lysates (*n* = 5 for *IR*^fl/fl^ and iFIRKO). (**c**) Time course of RER ($\dot{V}{\mathrm{CO}}_2$/$\dot{V}{\mathrm{O}}_2$) during induction of IR knockout by tamoxifen (arrows indicate tamoxifen gavage) (*n* = 5 for *IR*^fl/fl^; *n* = 6 for iFIRKO). (**d**) Time course of NEFA concentration in mice fed ad libitum (*n* = 6–13 for *IR*^fl/fl^; *n* = 6 for iFIRKO). (**e**) Total fat mass measured by EchoMRI 0, 7 and 28 days after tamoxifen (*n* = 8 for *IR*^fl/fl^; *n* = 6 for iFIRKO). (**f**) iBAT, ingWAT and epiWAT wet weight 5 days (5 d) or 28 days (28 d) after tamoxifen (*n* = 11 for *IR*^fl/fl^ (5 d); *n* = 6 for *iFIRKO* (5 d); *n* = 8 for *IR*^fl/fl^ (28 d); *n* = 7 for iFIRKO (28 d)). (**g**) Plasma leptin concentration measured with ELISA 3, 5 and 28 days after tamoxifen (*n* = 5–6 for *IR*^fl/fl^; *n* = 5–7 for iFIRKO). (**h**) Plasma insulin concentration measured by ELISA 7 and 28 days after tamoxifen (*n* = 6–7 for *IR*^fl/fl^; *n* = 6–8 for iFIRKO). (**i**) IPGTT in 4 h-fasted iFIRKO and *IR*^fl/fl^ control mice, 7 days after tamoxifen administration (*n* = 8 for *IR*^fl/fl^; *n* = 6 for iFIRKO). (**j**) Amount of triacylglycerol in soleus muscle, 2 weeks after tamoxifen administration (*n* = 6 for *IR*^fl/fl^; *n* = 7 for iFIRKO). (**k**) Glucose infusion rate during steady state of hyperinsulinaemic–euglycaemic glucose clamping in iFIRKO and *IR*^fl/fl^ littermate controls (*n* = 9 for *IR*^fl/fl^; *n* = 7 for iFIRKO). (**l**) Endogenous glucose production rate under basal and insulin-stimulated conditions (*n* = 5 for *IR*^fl/fl^; *n* = 4 for iFIRKO). (**m**) Uptake of 14C-glucose per mg tissue into soleus muscle and ingWAT (*n* = 5–6 for *IR*^fl/fl^; *n* = 6 for iFIRKO). In (**k**–**m**) hyperinsulinaemic–euglycaemic clamps were performed 7 days after tamoxifen administration. Data are mean ± SEM. Student’s *t* test: *p < 0.05, **p < 0.01, ***p < 0.001 for iFIRKO vs *IR*^fl/fl^ or as shown. In (**i**) difference is significant for all time points below the line. d, days; EGP, endogenous glucose production rate; GIR, glucose infusion rate; M. soleus, soleus muscle; TAG, triacylglycerol; Tam, tamoxifen

In order to investigate the effect of adipose tissue insulin resistance on whole-body metabolism, we studied inducible adipocyte-specific IR knockout mice, also called iFIRKO mice (*IR*^fl/fl^ × *Adipoq-*CreERT2). One week after tamoxifen gavage, iFIRKO mice showed a marked reduction of IR-β protein levels in adipose tissue depots (inguinal white adipose tissue [ingWAT], interscapular brown adipose tissue [iBAT] and epididymal white adipose tissue [epiWAT]), but not in liver (Fig. 1a, b). Three days after tamoxifen treatment, we measured $\dot{V}\mathrm{C}{\mathrm{O}}_2$ and $\dot{V}{\mathrm{O}}_2$ and observed a decrease in the RER in iFIRKO mice (Fig. 1c), while $\dot{V}{\mathrm{O}}_2$ and activity levels of mice remained unchanged (ESM Fig. 1a, b). Thyroid hormones are supposed to be plasma markers for increased basal metabolic rates. We found an increase in T_4_, which is the precursor of T_3_, in iFIRKO mice (ESM Fig. 1c). To resolve the timeline, we measured plasma NEFA concentrations in randomly fed mice after induction of the IR knockout. Coinciding with the change in RER, NEFA concentration increased by 40% in plasma of iFIRKO mice 3 days after induction (Fig. 1d). One week of IR ablation in adipocytes did not change the overall fat mass, while 4 weeks post induction we observed a significant change in adipose tissue mass (Fig. 1e). Interestingly, at day 5 of IR knockout in adipocytes, a reduction in iBAT and ingWAT but not in epiWAT mass was seen (Fig. 1f). The reduction of ingWAT mass was accompanied by reduced *Lep*, *Adipoq* and *Retn* gene expression in this fat depot (ESM Fig. 1d) in conjunction with a 70% reduction of plasma leptin levels (Fig. 1g), while plasma insulin concentration increased fourfold in iFIRKO mice at day 7 (Fig. 1h). At 7 days of IR knockout, we performed an IPGTT to avoid confounding effects from gut glucose absorption and an ITT, which showed that iFIRKO mice were glucose intolerant and insulin resistant (Fig. 1i and ESM Fig. 1e).

Four weeks after knockout, a normalisation of insulin and leptin plasma concentrations (Fig. 1g, h) and adipokine gene expression (ESM Fig. 1d) was observed. iFIRKO mice had half as much fat as the *IR*^fl/fl^ control mice (Fig. 1e) and the decrease in fat mass correlated with an increase in lean mass (ESM Fig. 1f), while total body mass remained unchanged (ESM Fig. 1g). During the same course of time, epiWAT mass was also reduced (Fig. 1f). The increase in liver mass could only account for a minor part of lean mass changes (ESM Fig. 1f). Unexpectedly, we observed no significant increase in liver triacylglycerol content (ESM Fig. 1i), while muscle triacylglycerol was reduced in iFIRKO mice (Fig. 1k and ESM Fig. 1j).

To further dissect the pathophysiology of adipose-specific deletion of IR, we performed hyperinsulinaemic–euglycaemic clamps in iFIRKO mice and their littermate *IR*^fl/fl^ controls. At 7 days, we observed a glucose infusion rate of close to zero in iFIRKO mice (Fig. 1k and ESM Fig. 1k, l). Furthermore, iFIRKO mice demonstrated higher hepatic glucose output (Fig. 1l) and lower glucose uptake by the soleus muscle and the ingWAT (Fig. 1m and ESM Fig. 1m). Blood glycerol concentrations remained unchanged during the course of IR knockout (ESM Fig. 1n, o).

### Fat loss after IR knockout induction in adipocytes is chronic, but not caused by adipocyte loss

**Fig. 2:**
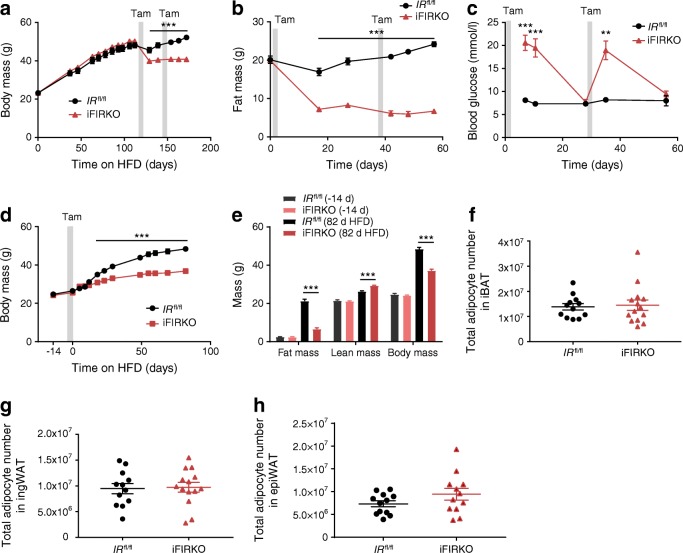
Diet-induced obese mice chronically lose fat tissue mass by induction of adipose tissue-specific IR knockout while, in parallel, mice are protected from diet-induced obesity when knockout is carried out before the initiation of HFD feeding. (**a**) Body mass and (**b**) fat mass of HFD-induced obese mice before and after induction of IR knockout by tamoxifen in adipose tissue (*n* = 5–16 for *IR*^fl/fl^; *n* = 5–16 for iFIRKO). (**c**) Ad libitum-fed blood glucose after two consecutive periods of induction of IR knockout in HFD-induced obese mice (*n* = 4–7 for *IR*^fl/fl^; *n* = 5–9 for iFIRKO). (**d**) Effect of HFD on body mass of lean mice after induction of adipose tissue-specific IR deletion (*n* = 11 for *IR*^fl/fl^; *n* = 5 for iFIRKO). (**e**) Evaluation of body composition and EchoMRI measurements of lean and fat mass in iFIRKO and *IR*^fl/fl^ littermate controls before and after an 11 week HFD challenge. Adipose tissue-specific IR knockout was induced at the initiation of the HFD challenge (*n* = 11 for *IR*^fl/fl^; *n* = 5 for iFIRKO; d, days). (**f**–**h**) Total adipocyte number in whole iBAT, ingWAT and epiWAT depots 1 week after tamoxifen induction, as measured by quantitative PCR for the evaluation of all cells that demonstrated the loxPStoploxP-tdRFP recombination (*n* = 12 for *IR*^fl/fl^; *n* = 14 for iFIRKO). Grey bars in (**a**–**d**) indicate time period of tamoxifen gavage. Data are mean ± SEM. Student’s *t* test: **p < 0.01, ***p < 0.001 for iFIRKO vs *IR*^fl/fl^ or as shown. In (**a**, **b**, **d**) difference is significant for all time points below the line. d, days; Tam, tamoxifen

To study the effect of adipocyte-specific IR ablation on adipose tissue mass under obesogenic conditions, we induced the knockout after 16 weeks of HFD (Fig. 2). In diet-induced obese mice, the genetic ablation of IR strongly reduced body weight (Fig. 2a). We observed that following IR knockout in adipocytes, 10.4 g loss of body weight was due to a 12.7 g loss in fat mass (Fig. 2b), which was paralleled by a reduction in the size of all adipose depots analysed (ESM Fig. 2a). Along with this, lean mass increased in iFIRKO mice (ESM Fig. 2b). Similar to previous findings in chow-fed iFIRKO mice, leptin concentration was reduced in diet-induced obese mice after induction of IR knockout in adipocytes by 70% (ESM Fig. 2c), and liver weight was increased by 0.8 g 4 weeks after induction of IR knockout in adipocytes (ESM Fig. 2d).

Subsequently, we sought to metabolically characterise already obese iFIRKO and *IR*^fl/fl^ mice that were on an HFD. We observed that obese iFIRKO mice developed extreme hyperglycaemia 1 week after induction of IR knockout in adipocytes and returned to normoglycaemia at 4 weeks, a pattern that was similar to the aforementioned development of glucose levels in chow-fed iFIRKO mice (Fig. 2c). Additionally, 1 week after induction of IR knockout in adipocytes, obese iFIRKO mice were insulin resistant (ESM Fig. 2e). While obese iFIRKO mice showed no difference in ambulatory activity (ESM Fig. 2f), the development of whole-body insulin resistance coincided with a reduced RER (ESM Fig. 2g, h). To our surprise, IR knockout in adipocytes of obese mice led to an increased $\dot{V}{\mathrm{O}}_2$ rate, which lasted from day 3 to day 10 (ESM Fig. 2i, j).

Because blood glucose levels in iFIRKO mice normalised 4 weeks after induction of IR knockout in adipocytes, while fat mass did not, we induced the knockout for a second time in the same mouse cohort in order to test whether fat mass could be reduced even further. Interestingly, renewed ablation of IR further reduced fat mass (Fig. 2b). Furthermore, the second induction of knockout led to the development of another hyperglycaemic phase, which again reversed within 4 weeks (Fig. 2c). This second knockout induction did not lead to liver mass alterations in hyperglycaemic iFIRKO mice, and only when normoglycaemia had already developed was an additional increase of liver mass observed. This indicates that normalisation of blood glucose levels might be independent of ectopically stored fat in the liver (ESM Fig. 2d).

In a different experimental approach, we induced IR knockout in adipocytes of adult chow-fed mice and subsequently changed their diet to an obesogenic HFD, to test the potential of re-growth of adipose tissue after it was rendered insulin resistant. Because of the inducible character of our adipocyte-specific insulin-resistant mouse model, newly differentiated cells will contain an unrecombined *IR* gene once tamoxifen is washed out. Starting at equal body and fat mass before the induction of knockout, iFIRKO mice gained less weight on HFD than *IR*^fl/fl^ control mice (Fig. 2d) and stayed protected from the obesogenic effect of HFD throughout week 12 (Fig. 2e). The difference in fat mass was even higher than that of body weight, because the lean mass was increased in iFIRKO mice (ESM Fig. 2b).

To address the question of whether IR knockout in adipocytes leads to their loss, we applied a new mouse model specifically generated to count adipocyte cells in iFIRKO mice. This triple-transgenic model, called the iFIRKO-chaser mouse, was created by combining the transgenes *Adipoq*-CreERT2 and *IR*^fl/fl^ with the floxed stop tdRFP allele (loxPStoploxP-tdRFP). After quantification of the recombination events in all cells of the adipose tissue depots iBAT, ingWAT and epiWAT, we could demonstrate that, 1 week after the induction of IR knockout, the total adipocyte number remained unchanged between *IR*^fl/fl^ control and iFIRKO mice (Fig. 2f–h). This suggests that adipose tissue mass reduction is due to lipid content reduction per adipocyte and not to disappearance of adipocytes.

### Antioxidants improve glucose intolerance of iFIRKO mice

**Fig. 3:**
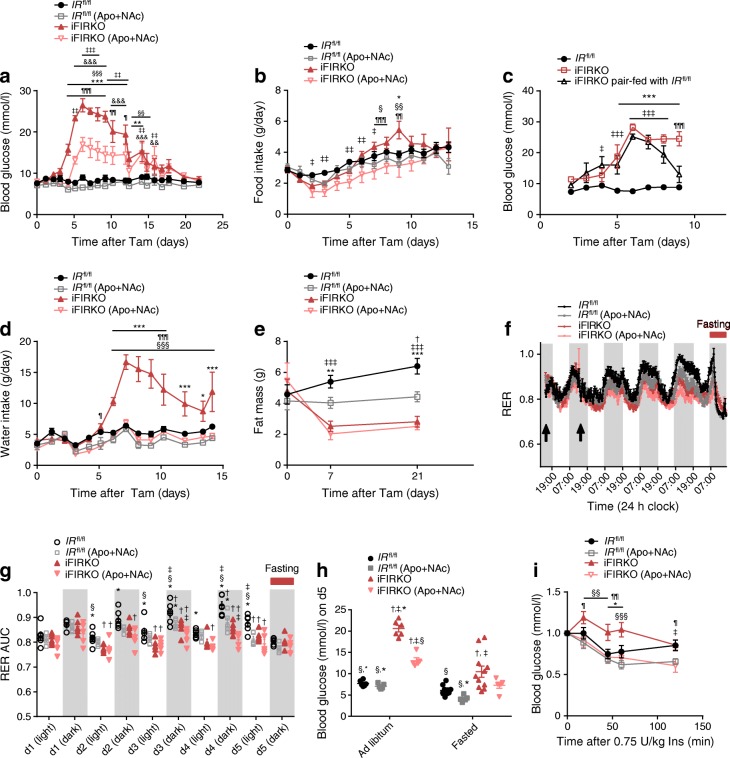
Supplementation of drinking water with apocynin (Apo, 40 mmol/l) and NAc (15 mmol/l) postpones and reduces hyperglycaemia, reduces food intake and enhances insulin sensitivity. (**a**) Blood glucose in ad libitum-fed iFIRKO and *IR*^fl/fl^ mice in either the presence or absence of Apo+NAc supplementation (*n* = 6 for *IR*^fl/fl^; *n* = 6 for *IR*^fl/fl^ (Apo+NAc); *n* = 6 for iFIRKO; *n* = 5 for iFIRKO (Apo+NAc)). (**b**) Recording of food intake in ad libitum-fed iFIRKO and *IR*^fl/fl^ mice in either the presence or absence of Apo+NAc supplementation (*n* = 6–13 for *IR*^fl/fl^; *n* = 6 for *IR*^fl/fl^ (Apo+NAc); *n* = 6 for iFIRKO; *n* = 5–12 for iFIRKO (Apo+NAc)). (**c**) Blood glucose from pair-fed iFIRKO mice upon pair-feeding with IR^fl/fl^; ad libitum-fed iFIRKO mice were used as controls (*n* = 7 for *IR*^fl/fl^; *n* = 4 for iFIRKO ad libitum-fed; *n* = 6 for iFIRKO pair-fed). (**d**) Water intake in ad libitum-drinking iFIRKO and *IR*^fl/fl^ mice in either the presence or absence of Apo+NAc supplementation (*n* = 6 for *IR*^fl/fl^; *n* = 6 for *IR*^fl/fl^ (Apo+NAc); *n* = 6 for iFIRKO; *n* = 5 for iFIRKO (Apo+NAc)). (**e**) Fat mass measured with EchoMRI in iFIRKO and *IR*^fl/fl^ mice either in the presence or absence of Apo+NAc supplementation (*n* = 6 for *IR*^fl/fl^; *n* = 6 for *IR*^fl/fl^ (Apo+NAc); *n* = 6 for iFIRKO; *n* = 5 for iFIRKO (Apo+NAc)). (**f**) RER ($\dot{V}{\mathrm{CO}}_2$/$\dot{V}{\mathrm{O}}_2$) time course (adipose tissue-specific IR knockout was induced where indicated with the arrows) and (**g**) RER AUC of dark/light cycle for iFIRKO and *IR*^fl/fl^ mice in either the presence or absence of Apo+NAc supplementation (*n* = 6 for *IR*^fl/fl^; *n* = 5 for iFIRKO); d, day. (**h**) Blood glucose levels in ad libitum-fed and fasted iFIRKO and *IR*^fl/fl^ mice in either the presence or absence of Apo+NAc supplementation (*n* = 6–11 for *IR*^fl/fl^; *n* = 5 for *IR*^fl/fl^ (Apo+NAc); *n* = 6–11 for iFIRKO; *n* = 5 for iFIRKO (Apo+NAc). (**i**) ITT using 0.75 U insulin per kg body mass (*n* = 6–11 for *IR*^fl/fl^; *n* = 5 for *IR*^fl/fl^ (Apo+NAc); *n* = 5 for iFIRKO; *n* = 6–11 for iFIRKO (Apo+NAc)). Data are mean ± SEM. Two-way ANOVA with Tukey’s multiple comparisons test: in (**a**, **b**, **d**, **e**, **g–i**) ^†^*p* < 0.05 for *IR*^fl/fl^ vs IR^fl/fl^ (Apo+NAc); ^*^p < 0.05, ^**^p < 0.01, ^***^p < 0.001 for *IR*^fl/fl^ vs iFIRKO; ^‡^*p* < 0.05, ^‡‡^*p* < 0.01, ^‡‡‡^*p* < 0.001 for *IR*^fl/fl^ vs iFIRKO (Apo+NAc); ^§^*p* < 0.05, ^§§^*p* < 0.01, ^§§§^*p* < 0.001 for *IR*^fl/fl^ (Apo+NAc) vs iFIRKO; ^¶^*p* < 0.05, ^¶¶^*p* < 0.01, ^¶¶¶^*p* < 0.001 for iFIRKO vs iFIRKO (Apo+NAc), ^&&^*p* < 0.01, ^&&&^*p* < 0.001 for *IR*^fl/fl^ (Apo+NAc) vs iFIRKO (Apo+NAc). In (**c**) ^***^p < 0.001 for *IR*^fl/fl^ vs iFIRKO; ^‡^*p* < 0.05, ^‡‡‡^*p* < 0.001 for *IR*^fl/fl^ vs iFIRKO pair-fed; ^¶¶¶^*p* < 0.001 for iFIRKO vs iFIRKO pair-fed. In (**a**–**d** and **i**) difference is significant for all time points below the line. d, day; Ins, insulin; Tam, tamoxifen

To elucidate the cause of hyperglycaemia in the iFIRKO animals, we analysed the development of glucose levels in a time course experiment, in vivo. We could show that hyperglycaemia development started 4 days after the induction of IR knockout in adipocytes (Fig. 3a), which notably is 1 day after the observed switch in energy substrate from glucose to fat. More specifically, the ad libitum random-fed blood glucose concentration increased from 8.96 ± 0.3 mmol/l in *IR*^fl/fl^ control mice to 26.5 ± 1.6 mmol/l in iFIRKO mice, 6 days after the induction of IR knockout time point when the peak of hyperglycaemia was observed. Throughout the time course experiment, hyperglycaemia (>19 mmol/l) in iFIRKO mice persisted for 7 days (until day 12 post induction of IR knockout) and subsequently decreased for 3 days, until similar levels were observed compared with *IR*^fl/fl^ control littermates on day 15 of IR knockout induction.

Despite reduced circulating leptin levels, iFIRKO mice did not consume more food than *IR*^fl/fl^ control mice after induction of IR knockout in adipocytes (Fig. 3b). Only on day 9 could food consumption be linked to blood glucose levels. When measuring random-fed blood glucose levels in mice, which were pair-fed to *IR*^fl/fl^ littermate controls, we observed that adipocyte-specific IR deletion-induced hyperglycaemia resolved faster in pair-fed iFIRKO mice (Fig. 3c). With a 2 day delay after the development of hyperglycaemia, iFIRKO mice showed an increase in water consumption (Fig. 3d).

Since many studies have reported that oxidative stress can cause insulin resistance by modulating lipotoxicity, we sought to evaluate whether antioxidants could have a beneficial role in our adipocyte-specific insulin-resistant model. As an antioxidant treatment, we chose the combination of NAc and apocynin. When mice were treated with antioxidants 1 day before induction of IR knockout, the development of hyperglycaemia in the iFIRKO mice was delayed by 1 day. Furthermore, antioxidant treatment reduced the random-fed hyperglycaemia to 17.0 ± 1.7 mmol/l compared with 26.5 ± 1.6 mmol/l in the untreated iFIRKO mice 6 days post induction of IR knockout (Fig. 3a). Acute administration of apocynin alone could not reproduce the beneficial effect of both antioxidants (Apo+NAc cocktail) (ESM Fig. 3a, b). Another effect of the antioxidant cocktail on iFIRKO mice was the reduction of food intake by 40% (Fig. 3b). The increase in water intake, which developed in iFIRKO mice 7 days after IR knockout, was blunted by the antioxidant cocktail (Fig. 3d). The reduction of fat mass caused by IR knockout was unchanged by antioxidant treatment (Fig. 3e). Although antioxidant administration did not lead to a reduction of fat mass in *IR*^fl/fl^ control mice, it counteracted the increase in fat mass observed in *IR*^fl/fl^ control mice. Body mass was reduced in both iFIRKO and *IR*^fl/fl^ control mice by antioxidant treatment (ESM Fig. 3c). This reduction in body mass on day 7 of treatment is due to a comparable reduction in lean mass of both iFIRKO and *IR*^fl/fl^ mice (ESM Fig. 3d).

To delineate the adipose-specific IR knockout-induced hyperglycaemia as well as the improvement in glucose tolerance caused by antioxidant supplementation, we evaluated substrate preference and energy expenditure in iFIRKO and littermate *IR*^fl/fl^ control mice. As mentioned above, iFIRKO mice had a significantly lower RER compared with littermate controls (Fig. 1b). Surprisingly, we observed that antioxidant supplementation reduced RER in wild-type mice but not in iFIRKO mice (Fig. 3f, g). Most importantly, the *IR*^fl/fl^ control group that was not supplemented with antioxidants did not demonstrate any significant RER differences compared with the other groups after fasting (Fig. 3f, g). Physical activity was similar in all groups (ESM Fig. 3e) and both iFIRKO and *IR*^fl/fl^ mice supplemented with antioxidants showed reduced $\dot{V}{\mathrm{O}}_2$ compared with the non-supplemented controls (ESM Fig. 3f, g). Along with the $\dot{V}{\mathrm{O}}_2$ rate, energy expenditure was reduced in the antioxidant-treated mice (ESM Fig. 3h, i). Interestingly, while the $\dot{V}{\mathrm{O}}_2$ and $\dot{V}{\mathrm{CO}}_2$ rates remained unchanged after IR knockout in adipocytes, the supplementation of antioxidants reduced $\dot{V}{\mathrm{CO}}_2$ during the dark phase more strongly in iFIRKO mice than in *IR*^fl/fl^ controls (ESM Fig. 3j, k).

As we could show that supplementation of antioxidants in iFIRKO mice led to a reduction in daily food intake (Fig. 3b), we measured random-fed and fasted blood glucose levels in wild-type and iFIRKO mice, which were not supplemented with antioxidants. Notably, we observed that the effect of antioxidants of reducing hyperglycaemia was present only in the random-fed and not in the fasted iFIRKO animals (Fig. 3h). To investigate the effect of antioxidants on insulin sensitivity, we performed an ITT 1 week after the induction of IR knockout in adipocytes. Similar to our previous results, we observed that antioxidants improved insulin sensitivity in iFIRKO mice but had no significant effect in wild-type mice (Fig. 3i).

### Antioxidants reduce hyperphagia and improve glucose homeostasis in *ob/ob* mice

**Fig. 4:**
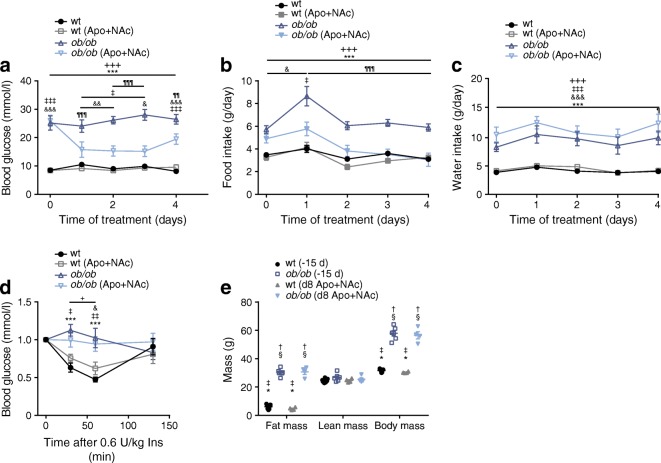
Supplementation of drinking water with apocynin (Apo, 40 mmol/l) and NAc (15 mmol/l) reduces hyperphagia and improves glucose homeostasis in leptin-deficient *ob/ob* mice. Apo+NAc supplementation was continuous and started on day 0 after measurement. (**a**) Blood glucose levels in ad libitum-fed *ob/ob* mice during treatment with antioxidants (*n* = 9 for wt; *n* = 8 for wt (Apo+NAc); *n* = 7 for *ob/ob*; *n* = 8 for *ob/ob* (Apo+NAc)). (**b**) Daily food intake in *ob/ob* mice during antioxidant treatment (*n* = 9 for wt; *n* = 8 for wt (Apo+NAc); *n* = 6 for *ob/ob*; *n* = 9 for *ob/ob* (Apo+NAc)). (**c**) Daily water intake in *ob/ob* mice during antioxidant treatment (*n* = 11 for WT; *n* = 11 for WT (Apo+NAc); *n* = 4 for *ob/ob*; *n* = 3 for *ob/ob* (Apo+NAc)). (**d**) ITT blood glucose over time, in min, after intraperitoneal injection of 0.6 U insulin per kg after 8 h fasting. Values normalised to blood glucose levels at injection (*n* = 6 for WT; *n* = 7 for WT (Apo+NAc); *n* = 7 for *ob/ob*; *n* = 8 for *ob/ob* (Apo+NAc)). (**e**) Body composition 15 days (−15 d) before and 8 days after (d8) antioxidant treatment (*n* = 7 for wt (−15 d); *n* = 4 for wt (d8 Apo+NAc); *n* = 6 for *ob*/*ob* (−15 d); *n* = 4 for *ob*/*ob* (d8 Apo+NAc). Data are mean ± SEM. Two-way ANOVA with Tukey’s multiple comparisons test: in (**a**–**d**) ^*^p < 0.05, ^***^p < 0.001 for wt vs *ob/ob*; ^‡^*p* < 0.05, ^‡‡^*p* < 0.01, ^‡‡‡^*p* < 0.001 for wt vs *ob/ob* (Apo+NAc); ^+^*p* < 0.05, ^+++^*p* < 0.001 for wt (Apo+NAc) vs *ob/ob*; ^¶^*p* < 0.05, ^¶¶^*p* < 0.01, ^¶¶¶^*p* < 0.001 for *ob/ob* vs *ob/ob* (Apo+NAc); ^&^*p* < 0.05, ^&&^*p* < 0.01, ^&&&^*p* < 0.001 for wt (Apo+NAc) vs *ob/ob* (Apo+NAc). In (**e**) ^†^*p* < 0.05 vs wt (−15 d); ^‡^*p* < 0.05 vs *ob*/*ob* (−15 d); ^§^*p* < 0.05 vs wt (d8 Apo+NAc); ^*^p < 0.05 vs *ob*/*ob* (d8 Apo+NAc). In (**a**–**d**) difference is significant for all time points below the line. d, day; Ins, insulin; WT, wild-type

After demonstrating a protective role of the antioxidant cocktail in hyperglycaemic iFIRKO mice, we evaluated whether this phenomenon is observed in other mouse models. Therefore, we examined the effect of supplementation of antioxidants in leptin-deficient *ob/ob* mice. At 12 weeks of age, *ob/ob* mice had developed hyperglycaemia (Fig. 4a), accompanied by hyperphagia (Fig. 4b) and polydipsia (Fig. 4c). We observed that after 24 h of antioxidant supplementation, there was already a significant reduction in the levels of random-fed blood glucose in *ob/ob* mice, compared with the control *ob/ob* mice which did not receive any antioxidant supplementation (Fig. 4a). In accordance with the effect of antioxidants in the iFIRKO mouse model, we observed that antioxidant supplementation did not acutely improve insulin sensitivity in this diabetic mouse model. (Fig. 4d). Therefore, we assume that regulation of food intake could be responsible for the observed beneficial hypoglycaemic effect of antioxidant supplementation. By measuring the daily food intake of *ob/ob* and wild-type mice, in combination with antioxidant supplementation, we confirmed that antioxidants significantly reduced food intake in *ob/ob* but not in wild-type mice (Fig. 4b and ESM Fig. 4a). Food over-consumption of heavily obese *ob/ob* mice (Fig. 4b) normalised to that of the control group by 2 days of treatment with antioxidant cocktail supplemented into the drinking water (Fig. 4b). We assume no taste aversion since antioxidant cocktail supplementation did not change daily water intake (Fig. 4c). Additionally, supplementation of apocynin alone could not reproduce the same effect as the administration of both antioxidants in parallel (ESM Fig. 4b, c). Finally, the short-term antioxidant supplementation did not demonstrate any effect on the fat or lean mass of *ob/ob* mice (Fig. 4e).

## Discussion

Based on the use of the new iFIRKO-tracer mouse model, we conclude that the iFIRKO mouse model does not resemble the typical lipoatrophic mouse models such as the FAT-ATTAC [19] or the A-Zip/F-1 mouse models [20], which exhibit a loss of adipocytes and ectopic deposition of lipids. The finding that adipose tissue can re-establish its storage function within a few weeks supports the notion of its high plasticity [21]. Importantly, in iFIRKO mice there seems to be no full compensation for the reduction of adipocyte size and mass of the various adipose tissue depots, because they are protected from excessive weight gain when mice are fed an obesogenic diet after the induction of IR knockout.

The adiponectin gene regulatory elements that we used to target adipocytes are currently the most specific known [22, 23]. The inducible nature of our iFIRKO mouse model allowed us to study different phases of the development of type 2 diabetes. At the start of the initial phase, insulin resistance could arise as a consequence of lipolysis-induced hyperinsulinaemia through lipotoxicity [24–27]. Our pair-feeding experiment indicates that hyperphagia of iFIRKO mice is not causative for the initial hyperglycaemia development, but rather that it contributes at a later phase. Results of hyperinsulinaemic–euglycaemic clamp experiments suggest that the subsequent development of hyperglycaemia in iFIRKO mice might be due to reduced hepatic insulin sensitivity of liver, which results in an increased endogenous glucose production [28]. One important conclusion is that lipids released from adipose tissue can be utilised by the respective tissues, which is supported by our finding of reduced lipid content in muscle, the observed switch in energy substrate and the reversibility of the type 2 diabetes symptoms in iFIRKO mice. While in lean iFIRKO mice an increase of T_4_ was detectable, in obese iFIRKO mice the increased lipid metabolising led to higher $\dot{V}{\mathrm{O}}_2$ rates. Further experiments at thermoneutrality or in the absence of Ucp1 could help to delineate the contribution of brown adipose tissue to this elevated basal metabolic rate.

One key molecular mechanism behind lipotoxicity is lipid-induced mitochondrial oxidative stress [13, 29]. Ethnological studies suggest that plants with antioxidant capacity can be used to treat diabetes-like symptoms [30, 31]. However, more recent clinical studies, which tested food supplementation with different antioxidants in the context of human diabetes, have questioned their beneficial effects [32, 33]. Unlike other studies [12, 34], we used a combination of the potent antioxidants NAc [35, 36] and apocynin [37, 38] to treat mice briefly before and while they were developing diabetes. The proposed targets of antioxidants are reactive oxygen species; however, as reactive oxygen species are very unstable molecules and oxidative stress is tightly regulated by antioxidant enzymes [39, 40], the exact contribution is hard to define. A few reports exist which demonstrate an improvement in insulin resistance in leptin-deficient *ob/ob* mice, while other studies supplementing food with antioxidants (e.g. apple polyphenol extracts or melatonin) have failed to report an effect on food intake [41, 42]. A blood glucose-lowering effect of apocynin, structurally related to vanillin [43], was reported in morbidly chronically obese KKAy mice [12]. In our system, acute treatment with apocynin reduced hyperglycaemia in neither iFIRKO mice nor *ob/ob* mice. The suggestion that the effect of the antioxidants NAc and apocynin is leptin independent is further supported by our observation that they mediate an effect during the early phase of diabetes development in iFIRKO mice in which leptin is almost absent.

Melatonin in its function as an antioxidant was reported to improve non-alcoholic fatty liver disease symptoms [42]. Additionally, the supplementation of apple polyphenol extracts reduced inflammation in *ob/ob* mouse liver, which was suggested as the site of its action [41]. We demonstrated here that iFIRKO mice are insulin resistant even under fasting conditions; however, as fasting lowered the RER of the control groups, we conclude that the substrate change from glucose to lipids observed after antioxidant treatment is not directly causal for the alterations of insulin resistance. Rather, we propose that the accumulation of oxidative damage over several days is required to cause insulin resistance. Unfortunately, the identification of factors that are secreted from tissues damaged by oxidative stress is beyond the scope of this study.

## Electronic supplementary material

ESM 1 (PDF 2.70 MB)

## Data Availability

Primary data are available upon request. Please contact the corresponding author.

## Abbreviations

ApoB: Apolipoprotein B
epiWAT: Epididymal white adipose tissue
HFD: High-fat diet
iBAT: Interscapular brown adipose tissue
iFIRKO: Inducible fat-specific insulin receptor knockout
ingWAT: Inguinal white adipose tissue
IR: Insulin receptor
NAc: *N*-acetylcysteine
qPCR: Quantitative PCR
RER: Respiratory exchange ratio
