# Supplementary material for: Antioxidants protect against diabetes by improving glucose homeostasis in mouse models of inducible insulin resistance and obesity
Source: Diabetologia. 2019 Jul 15;62(11):2094–105. doi: 10.1007/s00125-019-4937-7 (PMC6805816; doi:10.1007/s00125-019-4937-7)
Supplement: Supplementary file 1 — (PDF 2.70 MB) [file 125_2019_4937_MOESM1_ESM.pdf]

Tables

ESM Table 1 : Forward and revers primers sequences.

Gene	Forward primer sequence	Reverse primer sequence§
Tbp	5’-GAAGCTGCGGTACAATTCCAG-3’	5’-CCCCTTGTACCCTTCACCAAT-3’
Lep	5’-CAGGATCAATGACATTTCACACA-3’	5’-GCTGGTGAGGACCTGTTGAT-3’
Pparg2	5’-GCATGGTGCCTTCGCTGA-3’	5’-TGGCATCTCTGTGTCAACCATG-3’
Adipoq	5’-CGATTGTCAGTGGATCTGACG-3’	5’-CAACAGTAGCATCCTGAGCCCT-3’
Retn	5’-CTGTCCAGTCTATCCTTGCACAC-3’	5’-CAGAAGGCACAGCAGTCTTGA-3’

ESM Table 2: adipocyte quantification primers

primer	Forward primer sequence	Reverse primer sequence§
Recombined ^{fl}stop^{fl}tdRFP	5’- GCGCATGAACTCTTTGATGAC-3’	5’- TCGCGGTTGAGGACAAACTC -3’
ApoB	5’- GTCCAGGTTGAATCACGGGT -3’	5’- AGGATCCTGCAAGGTCAAGC -3’
pUC57 insert for standard curve	5’-CTTGAAGCGCATGAACTCTTTGATGACGTCCTCGGAG GAGGCCAGCATGGATCCAGCGCTAGCTTGGCTGGACGT AAACTCCTCTTCAGACCTAATAACTTCGTATAGCATACA TTATACGAAGTTATGCGGCCGACCGGTAAGCTTATCGAT ACCGTCGATCCCCACTGGAAAGACCGCGAAGAGTTTGT CCTCAACCGCGAGCTGTGGAGGTGGGGTCCAGGTTGAA TCACGGGTTCTTCAGCACAATGCACAGTTCTCCAATGAC CAAGAAGAAATACGGCTTGACCTTGCAGGATCCTTAGA CGGAGCCGGAGAACCTGCGTGCAATCCATCTTGTTCAA TGGCCGATCCCATGGCGGCACAGATGAATTCTTAATAA CTTCGTATAGCATACATTATACGAAGTTATGCGGCCG ACCGGTAAGCTTATCGATACCGTCGATCCCCACTGGAA AGACCGCGAAGAGTTTGTCCTCAACCGC-3’	

ESM Fig. 1

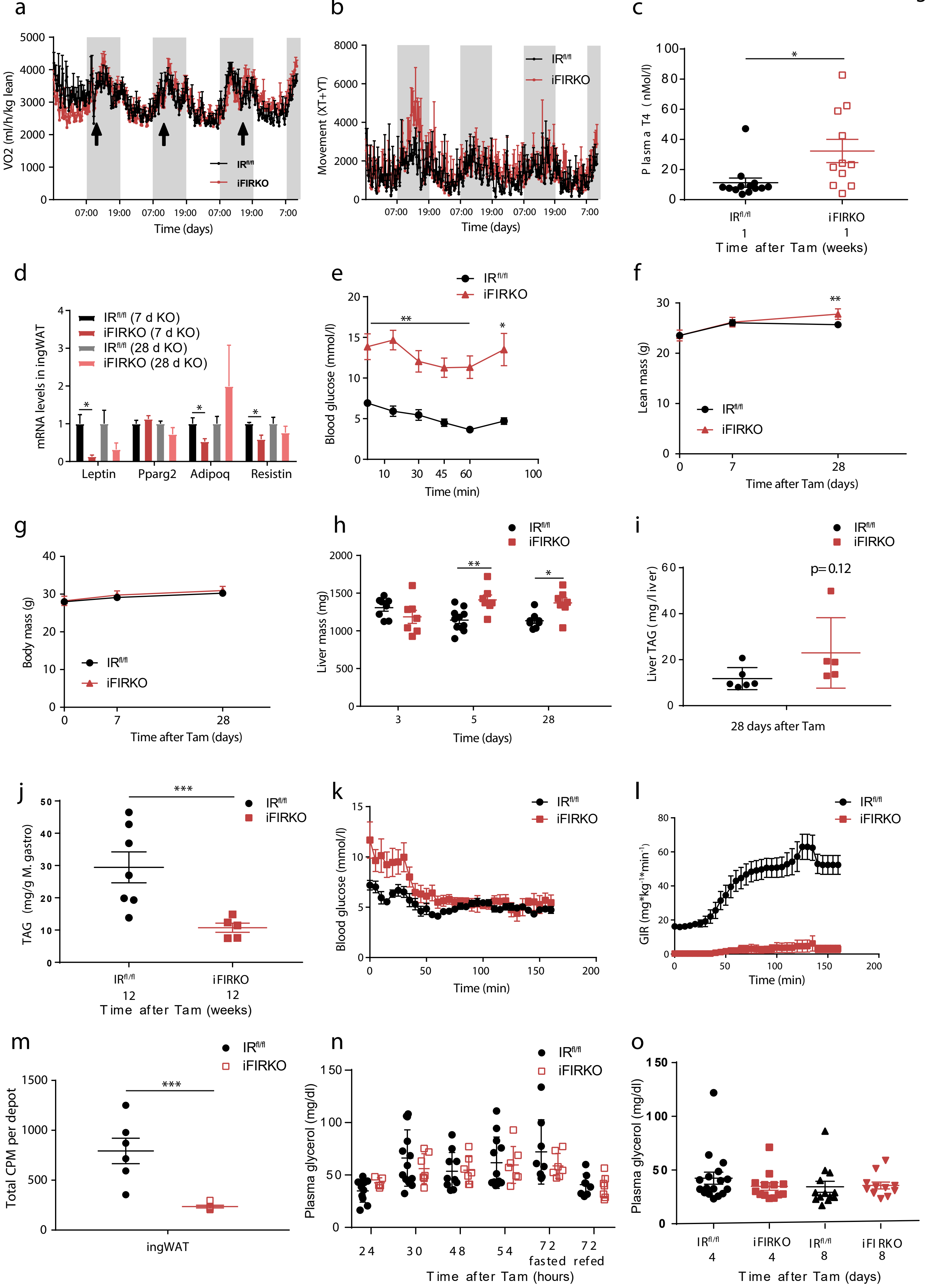

a
VO2 (ml/h/kg lean)
IR^fl/fl
iFIRKO
07:00 19:00 07:00 19:00 07:00 19:00 7:00
Time (days)
b
Movement (XT+YT)
IR^fl/fl
iFIRKO
07:00 19:00 07:00 19:00 07:00 19:00 7:00
Time (days)
c
Plasma T4 (nMol/l)
*
IR^fl/fl 1
iFIRKO 1
Time after Tam (weeks)
d
mRNA levels in ingWAT
IR^fl/fl (7 d KO)
iFIRKO (7 d KO)
IR^fl/fl (28 d KO)
iFIRKO (28 d KO)
Leptin
Pparg2
Adipoq
Resistin
e
IR^fl/fl
iFIRKO
Blood glucose (mmol/l)
Time (min)
f
Lean mass (g)
IR^fl/fl
iFIRKO
Time after Tam (days)
g
Body mass (g)
IR^fl/fl
iFIRKO
Time after Tam (days)
h
IR^fl/fl
iFIRKO
Liver mass (mg)
Time (days)
i
IR^fl/fl
iFIRKO
p=0.12
Liver TAG (mg/liver)
28 days after Tam
j
TAG (mg/g M. gastro)
IR^fl/fl
iFIRKO
IR^fl/fl 12
iFIRKO 12
Time after Tam (weeks)
k
IR^fl/fl
iFIRKO
Blood glucose (mmol/l)
Time (min)
l
IR^fl/fl
iFIRKO
GIR (mg*kg^-1*min^-1)
Time (min)
m
IR^fl/fl
iFIRKO
Total CPM per depot
ingWAT
n
IR^fl/fl
iFIRKO
Plasma glycerol (mg/dl)
24 30 48 54 72 fasted 72 refed
Time after Tam (hours)
o
Plasma glycerol (mg/dl)
IR^fl/fl 4
iFIRKO 4
IR^fl/fl 8
iFIRKO 8
Time after Tam (days)

ESM Fig. 1: Inducible fat-specific IR knockout (iFIRKO) does not affect oxygen consumption rate, but leads to increased lean mass and reduced adipokine mRNA expression. (a) metabolic cage measurement oxygen consumption (VO_2) and (b) movement (XT+YT) (n=5 for $IR^{fl/fl}$;6 for iFIRKO). (c) Plasma thyroxine (T4) concentration 1 week after Tam. (d) *Lep*, *Ppary2*, *Adipoq* and *Restn* qPCR of ingWAT mRNA (n=4 for $IR^{fl/fl}$ 7d KO;5 for iFIRKO 7d KO;7 for $IR^{fl/fl}$ 28d KO;4 for iFIRKO 28d KO). (e) ITT, blood glucose over time in minutes after intraperitoneal injection of 0.6 U insulin per kg after 8 hours fasting in iFIRKO and $IR^{fl/fl}$ control littermates (n=5-6 for $IR^{fl/fl}$;5-9 for iFIRKO). (f) Lean mass (g) body mass measured by EchoMRI 0 ,7 and 21 days after tamoxifen (n=8 for $IR^{fl/fl}$;6 for iFIRKO). (h) Liver mass 3, 5 and 28 days after tam (n=8-11;7). (i) Amount of TAG measured per liver 4 weeks after Tam gavage. (j) Amount of TAG in M. gastro. (k) Blood glucose concentration during hyperinsulinemic euglycemic glucose clamp (9 for $IR^{fl/fl}$;7 for iFIRKO). (l) Glucose infusion rate during clamp (n=9 for $IR^{fl/fl}$;7 for iFIRKO). (m) total counts per minute radiation uptake into ingWAT normalized to full depot mass. (n) Early time points blood glycerol concentration after induction of IR knockout in adipocytes. (o) Late time points blood glycerol concentration after induction of IR knockout in adipocytes. Mean+/-SEM. 2way ANOVA Turkey's multiple comparisons test one sign(e.g.*)=p<0.05, two signs(e.g.)**=p<0.01, three signs(e.g.***)=p<0.005, four signs (e.g.****)=p<0.001. TAG, triacylglycerol; Tam, tamoxifen; M. gastro, musculus gastrocnemius

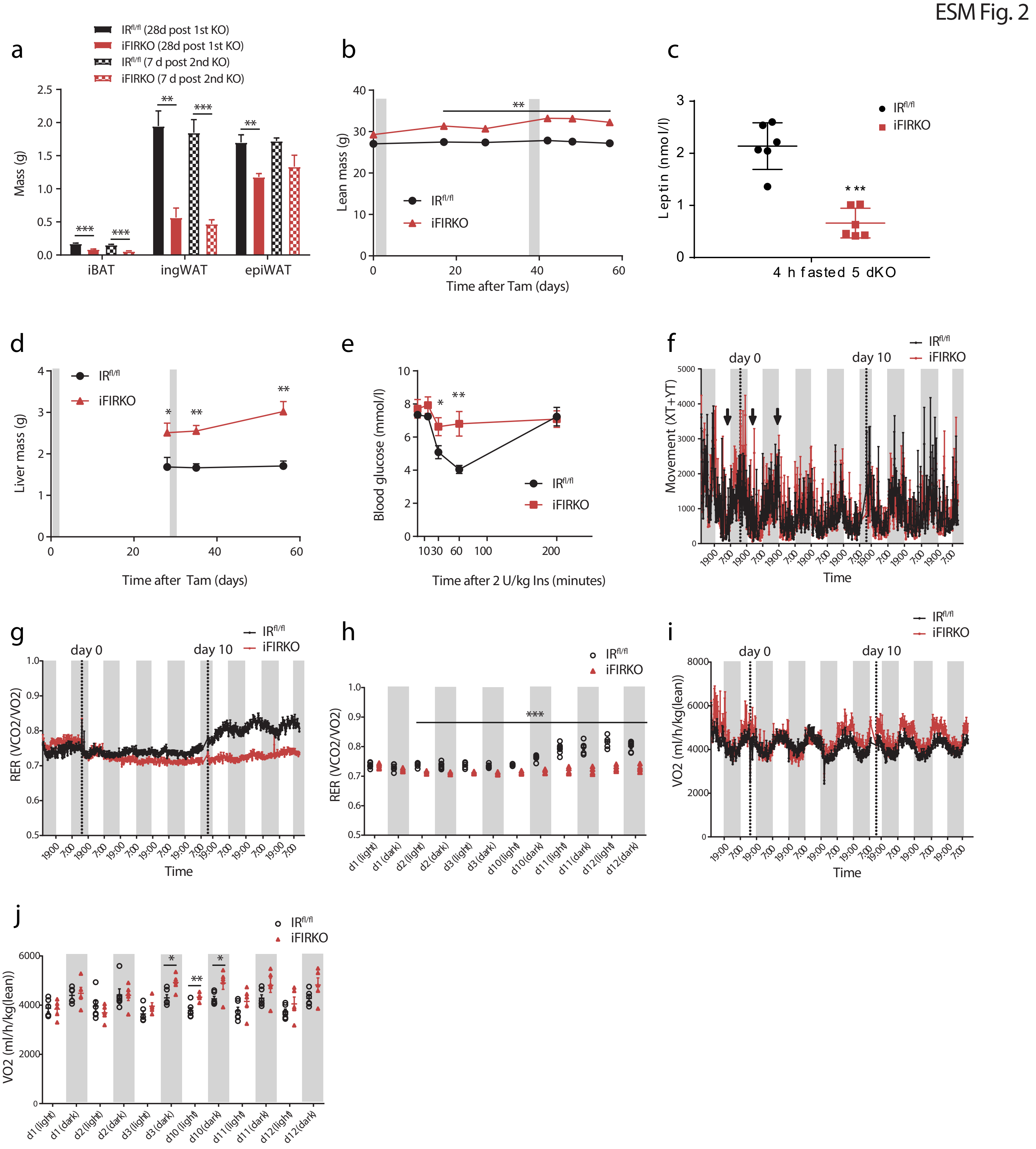
a
IR fl/fl (28d post 1st KO)
iFIRKO (28d post 1st KO)
IR fl/fl (7 d post 2nd KO)
iFIRKO (7 d post 2nd KO)
Mass (g)
iBAT
ingWAT
epiWAT
b
Lean mass (g)
Time after Tam (days)
IR fl/fl
iFIRKO
c
Leptin (nmol/l)
4 h fasted 5 dKO
IR fl/fl
iFIRKO
d
Liver mass (g)
Time after Tam (days)
IR fl/fl
iFIRKO
e
Blood glucose (mmol/l)
Time after 2 U/kg Ins (minutes)
IR fl/fl
iFIRKO
f
day 0
day 10
Movement (XT+YT)
Time
IR fl/fl
iFIRKO
g
day 0
day 10
RER (VCO2/VO2)
Time
IR fl/fl
iFIRKO
h
RER (VCO2/VO2)
IR fl/fl
iFIRKO
d1 (light)
d1 (dark)
d2 (light)
d2 (dark)
d3 (light)
d3 (dark)
d10 (light)
d10 (dark)
d11 (light)
d11 (dark)
d12 (light)
d12 (dark)
i
day 0
day 10
VO2 (ml/h/kg(lean))
Time
IR fl/fl
iFIRKO
j
VO2 (ml/h/kg(lean))
IR fl/fl
iFIRKO

ESM Fig.2: Diet induced obese mice chronically lose fat tissue mass and demonstrate reduced Leptin levels by induction of adipose tissue specific insulin receptor knockout while, in parallel, mice are protected from diet induced obesity and present with increased liver mass, when knockout is induced before HFD feeding started (a) Weight of adipose depots (iBAT, ingWAT and epiWAT) in HFD-induced obese mice after induction of IR knockout. Two different time points after the tam-induced knockout are shown: 28 days after the first and 7 days after the second tamoxiden induction (n=4 for $IR^{fl/fl}$ 28d post 1st KO;4 for iFIRKO 28d post 1st KO;4 for $IR^{fl/fl}$ 7d post 2nd KO;5 for iFIRKO 7d post 2nd KO). (b) Lean mass of HFD-induced obese mice before and after induction of IR knockout in adipose tissue (n=4-12 for $IR^{fl/fl}$;5-15 for iFIRKO). (c) Concentration of circulating leptin in HFD-induced obese mice measured 5 days after tam-induced IR knockout (n=6;6). (d) Liver mass of HFD-induced obese iFIRKO and $IR^{fl/fl}$ control mice at different time points after induction of IR knockout (n=4 for $IR^{fl/fl}$;4-5 for iFIRKO). (f) ITT in HFD-induced obese mice, 7 days after tam-induced insulin receptor adipose-tissue specific knockout (n=7 for $IR^{fl/fl}$;7 for iFIRKO). (g) Movement (XT+YT) measured in metabolic cage (n=6 for $IR^{fl/fl}$;5 for iFIRKO). (h) RER over time of the day (n=6;5). (i) Average RER for dark or light cycle (n=6 for $IR^{fl/fl}$;5 for iFIRKO). (j) oxygen consumption rate (VO_2) over time of the day (n=6;5). (k) Average VO_2 for dark or light cycle (n=6;5). Student's t-test *=p<0.05, **=p<0.01, ***=p<0.005. Tam, tamoxifen; ITT, insulin tolerance test; RER, respiratory exchange ration.

ESM Fig. 3

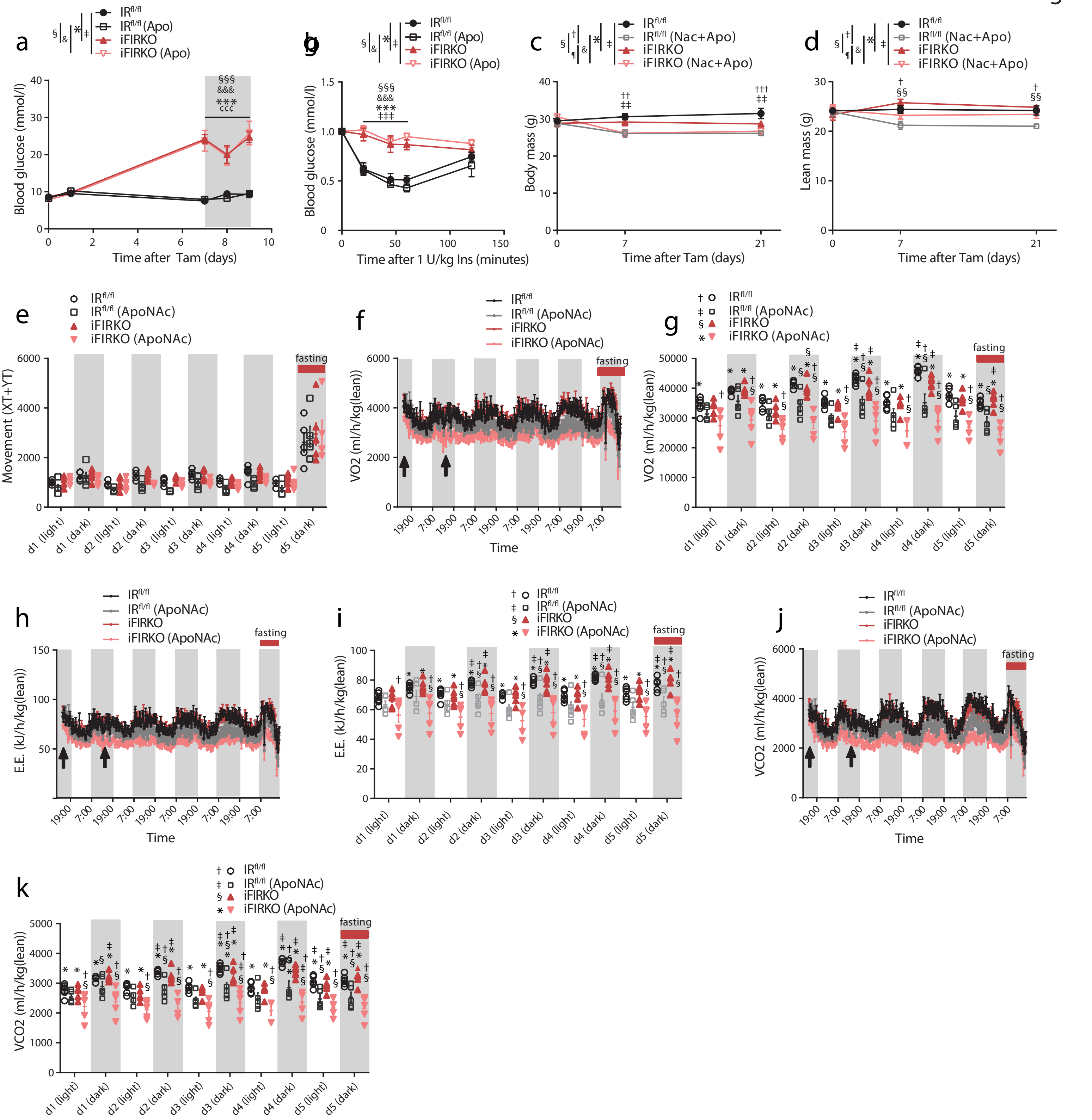

a
IRfl/fl
IRfl/fl (Apo)
iFIRKO
iFIRKO (Apo)
Blood glucose (mmol/l)
Time after Tam (days)
b
IRfl/fl
IRfl/fl (Apo)
iFIRKO
iFIRKO (Apo)
Blood glucose (mmol/l)
Time after 1 U/kg Ins (minutes)
c
IRfl/fl
IRfl/fl (Nac+Apo)
iFIRKO
iFIRKO (Nac+Apo)
Body mass (g)
Time after Tam (days)
d
IRfl/fl
IRfl/fl (Nac+Apo)
iFIRKO
iFIRKO (Nac+Apo)
Lean mass (g)
Time after Tam (days)
e
IRfl/fl
IRfl/fl (ApoNAc)
iFIRKO
iFIRKO (ApoNAc)
Movement (XT+YT)
fasting
d1 (light) d1 (dark) d2 (light) d2 (dark) d3 (light) d3 (dark) d4 (light) d4 (dark) d5 (light) d5 (dark)
f
VO2 (ml/h/kg(lean))
Time
g
VO2 (ml/h/kg(lean))
h
E.E. (kJ/h/kg(lean))
i
E.E. (kJ/h/kg(lean))
j
VCO2 (ml/h/kg(lean))
k
VCO2 (ml/h/kg(lean))

ESM Fig. 3: Supplementation of drinking water with apocynin (Apo, 40 mM) alone cannot reproduce the beneficial effects of the antioxidant cocktail (apocynin (Apo, 40mM) and (NAc, 15 mM)), whereas antioxidant cocktail supplementation reduces VO_2 and VCO_2 levels, both in iFIRKO and wild-type littermates. (a) Ad libitum blood glucose in iFIRKO and $IR^{fl/fl}$ mice either in the presence or absence of apocynin supplementation in the drinking water (n=6 for $IR^{fl/fl}$ – Apo+NAc;6 for $IR^{fl/fl}$ + Apo+NAc;5 for iFIRKO + Apo+NAc;6 for iFIRKO – Apo+NAc). (b) ITT, blood glucose over time in minutes after intraperitoneal injection of 1 U insulin/kg after 8 hours of fasting in iFIRKO and $IR^{fl/fl}$ mice either in the presence or absence of apocynin supplementation. (n=6 for $IR^{fl/fl}$ – Apo+NAc;6 for $IR^{fl/fl}$ + Apo+NAc;5 for iFIRKO + Apo+NAc;6 for iFIRKO – Apo+NAc). (c) Body mass and (d) lean mass measure with EchoMRI in iFIRKO and $IR^{fl/fl}$ mice either in the presence or absence of Apo+NAc supplementation (n=6 for $IR^{fl/fl}$ – Apo+NAc;6 for $IR^{fl/fl}$ + Apo+NAc;5 for iFIRKO +Apo+NAc;6 for iFIRKO – Apo+NAc). (e) Movement (XT+YT) measured inmetabolic cage (n=6;5;6;5). (f) oxygen consumption (VO_2) over time of the day. (g) VO_2 area under the curve for dark or light cycle (n=6 for IRfl/fl – Apo+NAc;5 for IRfl/fl + Apo+NAc;6 for iFIRKO + Apo+NAc;5 for iFIRKO – Apo+NAc). (h)E.E. over time of the day (n=6 for $IR^{fl/fl}$ – Apo+NAc;5 for $IR^{fl/fl}$ + Apo+NAc;6 for iFIRKO + Apo+NAc;5 for iFIRKO – Apo+NAc). (i) Average E.E. for dark or light cycle in iFIRKO and $IR^{fl/fl}$ mice (n=6;5;6;5). (j) VCO_2 over time of the day. (k) Average VCO_2 for dark or light cycle (n=6 for IRfl/fl – Apo+NAc;5 for IRfl/fl + Apo+NAc;6 for iFIRKO + Apo+NAc;5 for iFIRKO – Apo+NAc). Arrow symbol indicates time of tamoxifen gavage. Mean+/-SEM. 2way ANOVA Turkey's multiple comparisons test one sign(e.g.*)=p<0.05, two signs(e.g.)**=p<0.01, three signs(e.g.***)=p<0.005, four signs (e.g.****)=p<0.001. ITT, insulin tolerance test; EE, energy expenditure; VCO_2, CO_2 production. $^{+}p<0.05$, $^{++}p<0.01$, $^{+++}p<0.001$, $^{++++}p<0.0001$ for $IR^{fl/fl}$ vs. $IR^{fl/fl}$ (Apo+NAc), $^{*}p<0.05$, $^{**}p<0.01$, $^{***}p<0.001$, $^{****}p<0.0001$ for $IR^{fl/fl}$ vs. iFIRKO, $^{‡}p<0.05$, $^{‡‡}p<0.01$, $^{‡‡‡}p<0.001$, $^{‡‡‡‡}p<0.0001$ for $IR^{fl/fl}$ vs. iFIRKO (Apo+NAc), $^{§}p<0.05$, $^{§§}p<0.01$, $^{§§§}p<0.001$, $^{§§§§}p<0.0001$ for $IR^{fl/fl}$ (Apo+NAc) vs. iFIRKO, $^{¶}p<0.05$, $^{¶¶}p<0.01$, $^{¶¶¶}p<0.001$, $^{¶¶¶¶}p<0.0001$ for iFIRKO vs. iFIRKO (Apo+NAc), $^{\&}p<0.05$, $^{\&\&}p<0.01$, $^{\&\&\&}p<0.001$, $^{\&\&\&\&}p<0.0001$ for $IR^{fl/fl}$ (Apo+NAc) vs. iFIRKO (Apo+NAc).

ESM Fig. 4

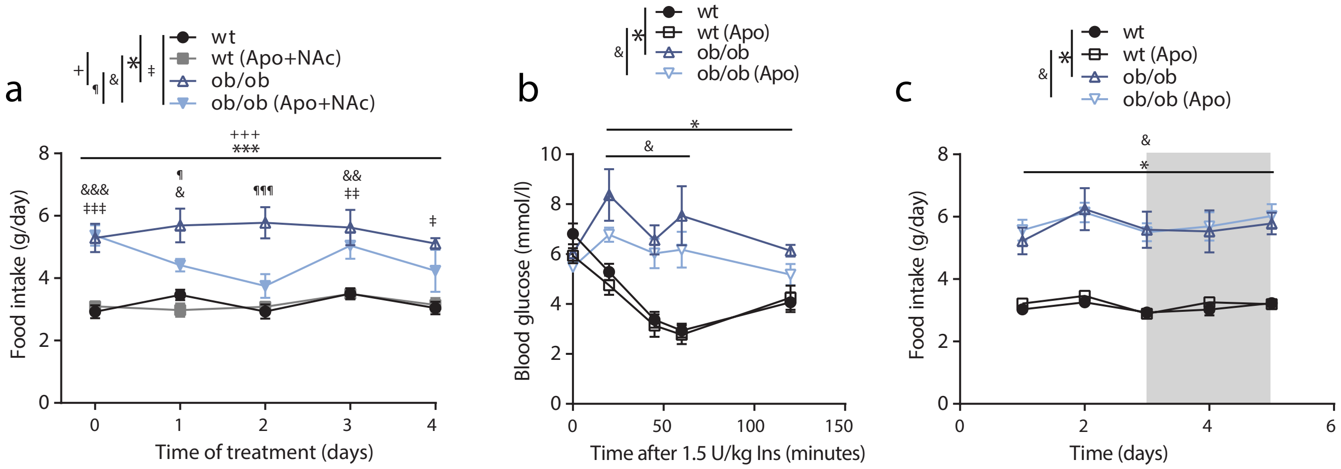
a
wt
wt (Apo+NAc)
ob/ob
ob/ob (Apo+NAc)
Food intake (g/day)
Time of treatment (days)
b
wt
wt (Apo)
ob/ob
ob/ob (Apo)
Blood glucose (mmol/l)
Time after 1.5 U/kg Ins (minutes)
c
wt
wt (Apo)
ob/ob
ob/ob (Apo)
Food intake (g/day)
Time (days)

ESM Fig. 4: Supplementation of drinking water with apocynin (Apo, 40 mM) and (NAc, 15 mM) reduced hyperphagia in female mice, whereas supplementation with apocynin (Apo, 40 mM) alone failed to reproduce this phenotype. (a) Daily food intake in wild-type and *ob/ob* female mice in the presence or absence of Apo+NAc administration (n=9 for wt – Apo+NAc;7 for wt + Apo+NAc;5 for ob/ob + Apo+NAc;4 for ob/ob - Apo+NAc). (b) ITT in wild-type and ob/ob mice in the presence or absence of apocynin administration (n=10 for wt – Apo+NAc;9 for wt + Apo+NAc;3 for ob/ob + Apo+NAc;4 for ob/ob - Apo+NAc). (c) Daily food intake in wild-type and ob/ob mice in the presence or absence of apocynin administration (n=11 for wt – Apo+NAc;11 for wt + Apo+NAc;3 for ob/ob + Apo+NAc;4 for ob/ob - Apo+NAc). 2way ANOVA Turkey’s multiple comparisons test one sign (e.g.*)=p<0.05, two signs(e.g.)**=p<0.01, three signs(e.g.***)=p<0.005, four signs (e.g.****)=p<0.001. ITT, insulin tolerance test. in (a) *p < 0.05, **p < 0.01, ***p < 0.001 for wt vs ob/ob; ‡p < 0.05, ‡‡p < 0.01, ‡‡‡p < 0.001 for wt vs ob/ob (Apo+NAc); +p < 0.05, +++p < 0.001 for wt (Apo+NAc) vs ob/ob; ¶p < 0.05, ¶¶p < 0.01, ¶¶¶p < 0.001 for ob/ob vs ob/ob (Apo+NAc); $^{\&}$p < 0.05, $^{\&\&}$p < 0.01, $^{\&\&\&}$p < 0.001 for wt (Apo+NAc) vs ob/ob (Apo+NAc). In (b-c) *p < 0.05 for wt vs ob/ob; $^{\&}$p<0.05 for wt (Apo) vs. ob/ob (Apo). In (a–c) difference is significant for all time points below the line. d, day; Ins, insulin; WT, wild-type
